# Novel Cell Culture Paradigm Prolongs Mouse Corneal Epithelial Cell Proliferative Activity *in vitro* and *in vivo*

**DOI:** 10.3389/fcell.2021.675998

**Published:** 2021-06-30

**Authors:** Xiaoya An, Guoliang Wang, Mengyi Jin, Xiaoping Zhou, Shubin Gao, Jingyao Chen, Peter S. Reinach, Zuguo Liu, Yuhua Xue, Cheng Li

**Affiliations:** ^1^Eye Institute & Affiliated Xiamen Eye Center, School of Pharmaceutical Sciences, School of Medicine, Xiamen University, Xiamen, China; ^2^Fujian Provincial Key Laboratory of Ophthalmology and Visual Science, Fujian Engineering and Research Center of Eye Regenerative Medicine, Xiamen, China; ^3^Yan’An Hospital Affiliated to Kunming Medical University, Kunming, China; ^4^School of Ophthalmology and Optometry, Eye Hospital, Wenzhou Medical University, Wenzhou, China

**Keywords:** cell culture, mouse corneal epithelial cells, EMT, small molecules, tissue engineering

## Abstract

It has been a long-standing challenge to obtain from cell cultures adequate amounts of mouse corneal epithelial cells (mCEC) to perform transplantation surgery. This limitation is attributable to the passage dependent declines in their proliferative activity. We describe here development of a novel 6C medium that contains six different modulators of different signaling pathways, which control proliferative mCEC activity. Its usage shortens the time and effort required to obtain epithelial sheets for hastening healing of an epithelial wound in an experimental animal model. This serum-free 6C medium contains:Y27632, forskolin, SB431542, DAPT, IWP-2, LDN-193189 and also DermaLife K keratinocyte calcium. Their inclusion inhibits rises in four specific markers of epithelial mesenchymal transdifferentiation:*ZEB1/2*, *Snail*, β*-catenin* and α*-SMA*. This medium is applied in a feeder-free air-lifted system to obtain sufficient populations of epithelial progenitor cells whose procurement is facilitated due to suppression of progenitor epithelial cell transdifferentiation into epithelial-mesenchymal cells. Diminution of this decline in transdifferentiation was confirmed based on the invariance of *P63*, *K14*, *Pax6*, and *K12* gene expression levels. This cell culture technique is expected to facilitate *ex vivo* characterization of mechanisms underlying cell fate determination. Furthermore, its implementation will improve yields of progenitor mouse corneal epithelial cells, which increases the likelihood of using these cells as a source to generate epithelial sheets for performing transplantation surgery to treat limbal stem cell deficiency in a clinical setting. In addition, the novel insight obtainable from such studies is expected to improve the outcomes of corneal regenerative medicine.

## Introduction

The cornea is a convex and highly transparent tissue providing approximately 75% of the ocular refractive power needed for normal visual acuity (DelMonte and Kim, 2011). Its outward epithelial layer facing the tear film is unique because it is made up of non-keratinized squamous cells organized in 5–6 layers (Kinoshita et al., 2001). These properties are essential for maintaining corneal transparency. They provide a physical barrier against pathogenic infiltration and injury to layers beneath the tissue’s outer surface (Ouyang et al., 2014). Preservation of these tight junctional and barrier functions is dependent on the ability of the epithelial cells to undergo continuous renewal to replace terminally differentiated cells in the uppermost layers. Otherwise, failure to rapidly restore tight junctional integrity increases the likelihood that environmental pathogens will enter into the cornea interior. If the outer layer barrier function is compromised, this can result in opacification, scarring, inflammation and neovascularization. Under normal conditions, the epithelial cells of the full thickness epithelium originate from a small stem cell population located in microenvironments at the junction between the corneal periphery and the adjoining conjunctiva. This unique domain is requisite for perpetuating these slow cycling cells which give rise to a continuous supply of proliferating progenitor cells. They are indispensable because they ultimately differentiate into unique cell types in the different epithelial cell layers. Therefore, studies focused on clarifying the underlying mechanisms controlling this chain of events are relevant for delineating the factors optimizing this renewal process.

Limbal stem cell deficiency is a clinical condition characterized by losses in functional corneal limbal stem cells (Ghareeb et al., 2020). Such malfunction can impair the ability of this layer to mediate net fluid transport from the stroma and establish effective barriers against the aforementioned stresses. If such functions are compromised, corneal swelling develops, which can lead to visual impairment and even blindness (Schwartz and Holland, 1998). Restoration of normal corneal epithelial function in a clinical setting may warrant corneal transplantation surgery. To optimize the outcome of this procedure, cell culture techniques are continuously being modified to improve the yields of viable epithelial cells used for this purpose. Limbal epithelial transplantation is predicted to mitigate the limited availability of eye bank corneas for transplantation surgery (Pellegrini et al., 1997). The success of this procedure is dependent on increasing the yields of proliferating precursor epithelial cells. Even though substantive progress has been made in this regard, it remains a stumbling block warranting further study to generate more readily available adequate populations of proliferating epithelial cells for corneal reconstruction surgery.

*In vivo* stabilization of corneal epithelial cell functionality and identity are highly dependent on precise spatiotemporal gene expression regulation by micro-environmental signals. It is known that yields of isolated primary corneal epithelial cells (CEC) are limited because they have trouble maintaining their core gene expression characteristics and specific functions. Substantial progress has been made to reverse these declines through developing innovative defined conditions for increasing CEC proliferation and adherence in culture. One alternative is the air lifted feeder-layer system which significantly increases the clonal formation efficiency of limbal stem and progenitor cells (Tseng et al., 1996). Feeder-conditioned media supplemented with serum inhibits spontaneous differentiation and allows indefinite self-renewal. On the other hand, downstream experimental results can be erratic owing to intrinsic variability in serum and feeder cell quality, as well as inconsistent manipulation techniques. Resolution of these types of problems are expected to hasten obtainment of larger yields of functional proliferating CEC that readily attach to the insert. Such an outcome is expected to increase insert yields needed for corneal reconstruction surgery, providing patients with a therapeutic option to counter losses in vision caused by severe limbal stem cell deficiency (Pellegrini et al., 1997; Schwab et al., 2000). Improved management of this condition will make it possible to better control this condition and make it less difficult for the patient to await until a suitable cornea becomes available for performing a keratoplasty (Ang et al., 2006). Alternatively, improved outcome of the corneal epithelial transplantation procedure may lessen the need to perform a corneal transplantation procedure in the future (Koizumi et al., 2001; Rama et al., 2010).

Compared with other common experimental species, primary mCEC are more difficult to culture *in vitro* because of their poor adherence, weak proliferative capacity and propensity to undergo epithelial-mesenchymal cells transition (EMT) (Kawakita et al., 2008). Small molecules with well-defined structures and target genes have been confirmed as effective tools for manipulating the fate, and the functional states of various stem cell and progenitor cell progeny. There has been significant progress in using small molecules to either sustain pluripotency or induce the differentiation of different cell types. Sun et al. succeeded in culturing human CEC in the absence of feeders by using Y-27632, a ROCK inhibitor (Sun et al., 2015). While this has been a successful strategy for different types of cell expansion, its supplementation failed to inhibit spontaneous EMT and thereby improve functional epithelial cell maintenance. Recently, Deng et al. reported that a reagent cocktail designated as 5C composed of forskolin, SB431542, DAPT, IWP-2 and LDN-193189 reduced the expression of EMT marker genes, effectively inhibited fibrosis of liver cells during *in vitro* culture and supported long-term Hepatitis B virus (HBV) infection *in vitro* (Xiang et al., 2019). Each compound contained in this 5C cocktail has been proven to improve corneal epithelium homeostasis maintenance (Nakamura et al., 1998; Li et al., 2013; Tsai et al., 2014; Choudhary et al., 2017; Lee et al., 2017; Kamarudin et al., 2018). In the current study, we compared on the mCEC culture the individual and combined effects of Y-27632 and 5C namely 6C.

Here, we show that this novel air lifting modified cell culture paradigm using the 6C cocktail to supplement the DermalLife medium increased the epithelial cell proliferation and sustained the expression patterns of progenitor cell function gene expression levels and suppressed EMT. This innovation lays a foundation for undertaking CEC plasticity research and it also provides a promising novel approach for improving corneal regenerative medicine.

## Materials and Methods

### Materials and Reagents

DermaLife K Keratinocyte Calcium-Free Medium Kit was purchased from Life Line (Oceanside, CA, United States). Dulbecco’s Modified Eagle Medium, Ham’s F-12 medium, fetal bovine serum (FBS), mouse epidermal growth factor (EGF), HEPES buffer, gentamicin, amphotericin B, trypsin-EDTA, TRIzol^®^ and the antibody of anti-ZO1 (339100) were purchased from Invitrogen (Carlsbad, CA, United States). Dispase II was purchased from Roche (Basel, Switzerland). Y-27632, Triton X-100, bovine serum albumin (BSA), insulin-transferrin-sodium selenite media supplement, hydrocortisone, cholera toxin and dimethyl sulfoxide (DMSO) were purchased from Sigma (St. Louis, MO, United States). Alexa Fluor 488− and Alexa Fluor 594-conjugated IgG were purchased from Life Technologies (Carlsbad, CA, United States). Forskolin, SB431542, DAPT, IWP-2, and LDN-193189 were purchased from Apexbio (Boston, MA, United States). The antibodies of rabbit anti-Ki67 (ab16667), rabbit anti-Pax6 (ab195045), rabbit anti-P63 (ab124762), rabbit anti-cytokeratin 14 (K14) (ab181595), and mouse anti-alpha smooth muscle actin (α-SMA) (ab18147) were purchased from Abcam (Cambridge, MA, United States). The antibodies specific for goat anti-cytokeratin 12 (K12) (sc-17101) and rabbit anti-β-catenin (sc7199) were purchased from Santa Cruz Biotechnology (Santa Cruz, CA, United States). The antibodies of rabbit anti-ZEB1 (a5600) and rabbit anti-Snail (a5243) were purchased from Abclonal (Boston, MA, United States). ExScript RT Reagent kit and SYBR Premix Ex Taq Kit were obtained from Takara Bio (Shiga, Japan). 12-well culture inserts used in this study were purchased from Millipore Corporation (Billerica, MA, United States).

### Cell Culture

C57BL/6 mice (6∼8 weeks) were obtained from the Experimental Animal Center of Xiamen University. All experimental procedures used for animals in ophthalmology and vision research are in compliance with the regulations of the Association for Research in Vision and Ophthalmology (ARVO) and have been approved by the Experimental Animal Ethics Committee of Xiamen University.

After mice were sacrificed by cervical dislocation, their eyeballs were harvested and incubated with 10 mg/ml dispase II at 4°C for 18 h. The corneal epithelium was isolated and digested with 0.05% Trypsin-EDTA at 37°C for 15 min. Cells were seeded at a density of 5,000 cells/cm^2^ in a 24-well dish. They were incubated at 37°C, 5% CO_2_ and 95% air, and the DermaLife K keratinocyte calcium and serum-free medium was replaced every 2 days. The cell morphology was observed and digital images were captured using a microscope (Olympus CKX53, Japan).

The experimental groups are as follows: Control group; Solvent group, containing DMSO (0.1%); Y-27632 group, containing Y-27632 (10 μmol/l); 5C group, containing Forskolin (20 μmol/l), SB431542 (10 μmol/l), DAPT (5 μmol/l), IWP-2 (0.5 μmol/l), LDN-193189 (0.1 μmol/l); 6C group, containing Forskolin (20 μmol/l), SB431542 (10 μmol/l), DAPT (5 μmol/l), IWP-2 (0.5 μmol/l), LDN-193189 (0.1 μmol/l), Y-27632 (10 μmol/l).

### Air-Lifted Exposure Culture

Mouse corneas were isolated with ophthalmic scissors, and then cut into petals with a blade. Next, explants were placed on inserts in 12-well plates containing DermaLife K keratinocyte calcium and serum-free medium with or without 6C for 1 week. To expose an epithelial explant to air, 0.5 ml of culture medium was added to each well. This amount was sufficient to only immerse the underlying stromal cells in medium. The corneal epithelium and epithelial single cells were harvested as described above. The planting area of the epithelial sheet was about one-tenth of the area of the insert, and the planting density of epithelial single cells was about 5,000/cm^2^. They were submerged in the medium until the cell density covered at least 90% of the inserts, and then the cultures were air lifted for 1 week.

### Cell Proliferation Assay

To evaluate the cell proliferative activity, the cells were trypsinized and harvested daily from three randomly selected wells and manually counted using a hemocytometer (Stewart et al., 2000). The protocol involved counting the number of cells in the upper left, lower left, upper right, and lower right grids of the hemocytometer. The cells in each well were counted 3 times, and the average cell density was used to generate a growth curve.

Crystal violet staining was also used to detect cell proliferation rates. After culturing for 1 week, cells were fixed with 4% paraformaldehyde at room temperature for 15 min, then washed three times with PBS for 5 min each and stained with 0.05% crystal violet at room temperature for 30 min, then washed three times with PBS for 5 min each and digital images were captured with a camera attached to a microscope.

### RNA Isolation, Reverse Transcription, and Quantitative Real-Time PCR (qRT-PCR)

The total cellular RNA was extracted with TRIzol and then reverse transcribed into cDNA using ExScript RT kit. qRT-PCR was performed with the SYBR Premix Ex Taq kit and the StepOne Real-Time PCR detection system (Applied Biosystems, Darmstadt, Germany) according to the manufacturer’s instruction. The amplification procedure included an initial denaturation step at 95°C for 10 min, followed by denaturation at 95°C for 10 s, and annealing and extension at 60°C for 30 s, for 40 cycles. Using β-actin as an internal control, the results of relative qRT-PCR were analyzed by the comparative C_T_ method (2-^ΔΔCt^) (Suzuki et al., 2000). Table 1 shows the primers used to amplify specific gene products obtained from mCEC cDNA.

**TABLE 1:** Primer sequences for qRT-PCR of indicated gene transcripts.

**Gene name**	**Sense**	**Antisense**
*Ki67*	CACTCCAAAGAAACCCACAA	CTCATCTGCTGCTGCTTCTC
*P63*	ATGTCACCGAGGTTGTGAAA	GAATTCAGTGCCAACCTGTG
*K14*	CCCACCTTTCATCTTCCCAATT	AAGCCTGAGCAGCATGTAGCAG
*K12*	AAACCGCAGACACCATCAGT	ATGAGACCACTTCGCCATTC
*Pax6*	AGTGTCTACCAGCCAATCCC	CATGGAACCTGATGTGAAGG
*ZEB1*	CCACTGTGGAGGACCAGAAT	GTGAGGCCTCTTACCTGTGT
*ZEB2*	AAGTACCGCCACGAGAAGAA	TTTGGTGCTGATCTGTCCCT
α*-SMA*	CTCCCTGGAGAAGAGCTACG	CGCTGACTCCATCCCAATGA
β*-catenin*	ACAAGAAGCGGCTTTCAGTC	CTGCAGTCTCATTCCAAGCC
β*-actin*	GAGACCTTCAACACCCCAGC	ATGTCACGCACGATTTCCC

### Colony-Forming Efficiency Assay

Clonal culture was performed according to Rheinwald and Green (R&G)’s method (Rheinwatd and Green, 1975). 3T3 fibroblasts were treated with mitomycin C (5 μg/ml) at 37°C for 3 h, and then digested with 0.25% Trypsin-EDTA at 37°C for 3 min, and terminated with SHEM medium (comprising an equal volume of HEPES-buffered DMEM containing bicarbonate and Ham’s F-12 medium, 2 ng/ml mouse EGF, 5 mg/ml insulin, supplemented with 5% FBS, 0.5% dimethyl sulfoxide, 5 mg/ml transferrin, 5 ng/ml selenium, 0.5 mg/ml hydrocortisone, 1 nM cholera toxin, 50 mg/ml gentamicin, and 1.25 mg/ml amphotericin B). 3T3 fibroblasts and mCEC (isolated as described above) were co-cultured at a density of 4.5 × 10^4^/cm^2^ and 2,000/cm^2^ in a 12-well dish.

### Histological Characteristics and Immunostaining

Corneal epithelial cell sheets were imbedded in opti-mum cutting temperature (OCT) compound, and cryostat sections (5 μm) were obtained. Cultured mCEC and frozen sections were rehydrated in PBS after being fixed in 4% paraformaldehyde for 20 min at room temperature, followed by incubation in 0.2% Triton X-100 for 10 min. After rinsing with PBS three times for 5 min each and preincubating with 2% BSA to block non-specific staining, the sections were incubated with primary antibodies overnight at 4°C with different dilutions [P63, β-catenin and α-SMA all at 1:100, Ki67, Pax6, K12, ZEB1, and Snail all at 1:200, K14 (1:1000), ZO-1 (5–10 μg/ml)]. After washing with PBS three times for 10 min each, cells or sections were incubated with Alexa Fluor-conjugated secondary antibodies for 1.5 h. After rinsing each section three times with PBS for 10 min, they were counterstained with DAPI and then mounted. A fluorescence microscope was used to view and analyze each section (Leica, Germany and Olympus FV1000MPE-B, Japan).

For morphological analysis, hematoxylin and eosin (H&E) staining was performed according to standard procedures. Digital images of representative areas were captured with the light microscope (Eclipse 50i, Nikon, Tokyo, Japan).

### Corneal Epithelial Wound Healing

The mice were anesthetized by intraperitoneal injection of pentobarbital (40 mg/kg). A corneal trephine with a diameter of 1.5 mm was used to make a circular mark on the cornea of the mouse, and the circular area was scraped off with a corneal spatula. Then the eyes were spotted with 6C solution and the solvent control group was set. The corneal epithelial defects were visualized at 0, 12, 24, and 36 h after injury by instillation of 0.5% fluorescein sodium and were examined and photographed with a camera mounted on a slit-lamp microscope (Haag-StreitAG, Switzerland).

### Statistical Analysis

ImageJ software was used to analyze the time dependent declines in fluorescein sodium staining area in a debrided region of the epithelium and crystal violet staining area of the cultured cells. Statistical analysis was performed with one-way analysis of variance analysis with GraphPad Prism 8.0.1 software. Unless otherwise stated in all figures, data are shown as mean ± SEM (*n* = 3). ^∗^*P* < 0.05, ^∗∗^*P* < 0.01, ^∗∗∗^*P* < 0.001, ^****^P < 0.0001.

## Results

### 6C Treatment Increases Adherence and Proliferation of Primary mCEC

**FIGURE 1:**
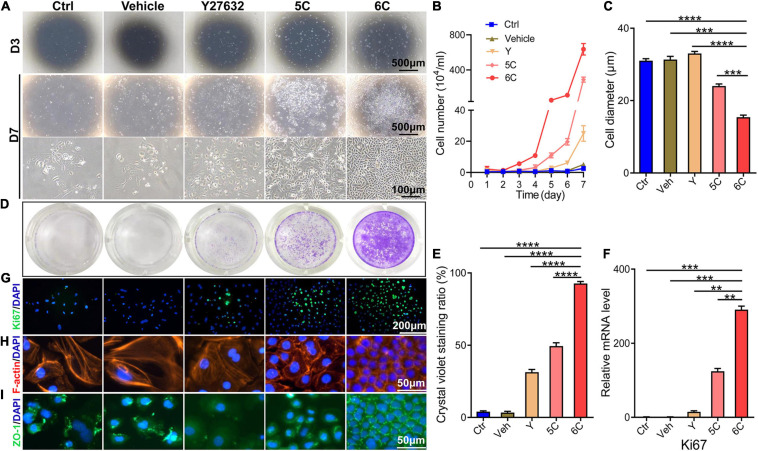
6C can promote the proliferation of primary mCEC. **(A)** Morphological comparison of mCEC cultured with different compositions. Images were taken at day 3 and day 7 post seeding. **(B)** Cell growth curve. **(C)** A statistical graph of the average diameter of the cells after 1 week of culture. **(D)** Crystal violet staining results after 1 week of cell culture. 6C group had the most stained area. **(E)** Statistical analysis of crystal violet staining results after 1 week of cell culture. (*n* = 5 biological replicates). **(F)** qRT-PCR analysis of *Ki67* gene expression (normalized to ctrl). **(G)** Ki67 (green) immunofluorescence staining. Nuclei were stained with DAPI (blue). **(H,I)** Immunofluorescence staining of F-actin (red) and ZO-1 (green). Nuclei were stained with DAPI (blue). The expression of F-actin and ZO-1 in 6C group was distributed on the cell membrane, and the connection between cells was tight, but the distribution of other treatment groups was scattered or missing. Data expressed as the means ± SEM from three separate experiments (**P < 0.01, ***P < 0.001, ****P < 0.0001).

It is well known that the mCEC were poorly adherent when cultured *in vitro* (Kawakita et al., 2004), but by the third day, most of the cells in the 6C treatment group adhered and the proliferation rate far exceeded that of the other four treatment groups (Figure 1A). Cell growth curves were generated to determine if culturing mCEC exposed to the 6C cocktail promoted their adherence and increased cell proliferation (Figure 1B). By day 7, the cells in both the control group and the vehicle group had irregular morphology and fell off. In both the 5C and the Y-27632-treated groups, the cells had a larger volume. It is apparent that the cells treated with 6C had a very regular oval morphological structure, and the cells were small and densely arranged (Figure 1C). The crystal violet cell staining area in the 6C group was greater than the other four treatment groups, indicating that 6C treatment enhanced cell proliferation (Figures 1D,E). Furthermore, Ki67 immunofluorescence staining and qRT-PCR analysis of proliferation related gene expression levels confirmed that 6C treatment promoted cell proliferation (Figures 1F,G). Fibrillar actin (F-actin) and tight junctional ZO-1 proteins play important roles in sustaining both cell adhesion and cell-cell tight junctional connectivity (Higbee and Hazlett, 1983; Ryeom et al., 2000). When the cells were treated with 6C *in vitro*, F-actin immunostaining appeared tight and regular, but its pattern in the other four treatment groups was less well defined and more irregular (Figure 1H). In addition, the expression of ZO-1 in the 6C-treated group was significant whereas in the other groups it was erratic (Figure 1I). These results show that 6C treatment promoted cell adhesion and intercellular adhesion.

### Effects of Each Component in 6C on the Growth of mCEC

**FIGURE 2:**
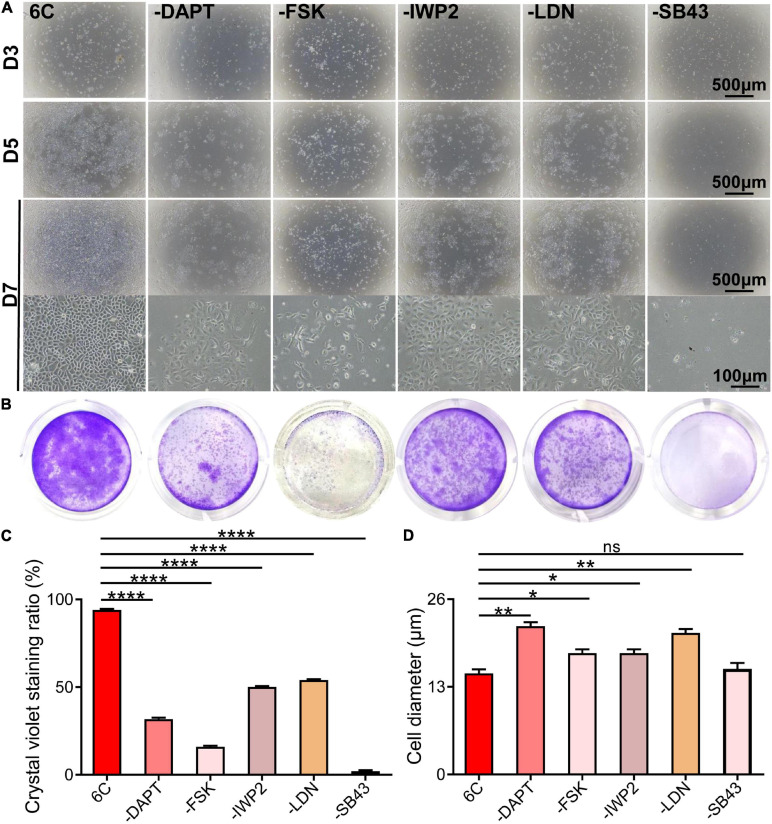
Effects of subtracting a compound on the proliferation of mCEC. **(A)** Morphological comparison of mCEC cultured with subtracting one compound from 6C or 6C, respectively (The group without Y-27632 has been described above). Images were taken at day 3, day 5 and day 7 post seeding. The cell proliferation rate of the 6C treatment group was much better than that of the treatment group minus any compound. **(B)** Crystal violet staining results after 1 week of cell culture. 6C group had the most stained area. **(C)** Statistical analysis of crystal violet staining results. Data expressed as the means ± SEM from three separate experiments (*P < 0.05, **P < 0.01, ****P < 0.0001, ns: no significance). **(D)** Statistical analysis of the diameters of the cells.

To determine if each component in the 6C cocktail is essential for the proliferation of mCEC, we compared the effects of a single component at a time on cell growth (Figure 2A). The results showed that omission of any one compound caused the cell proliferation to decline (Figures 2B,C) and compromised the cell morphology relative to that in the cultures treated with 6C cocktail (Figure 2D). In addition, the results showed that the cells in the treatment group without SB431542 almost lost their proliferative activity, which means that SB431542 plays an essential role in regulating the proliferation of mouse corneal epithelial cells. The growth of cells in the treatment group without FSK was more scattered and the cell status was poor.

### 6C Treatment Preserves Function of Primary mCEC

**FIGURE 3:**
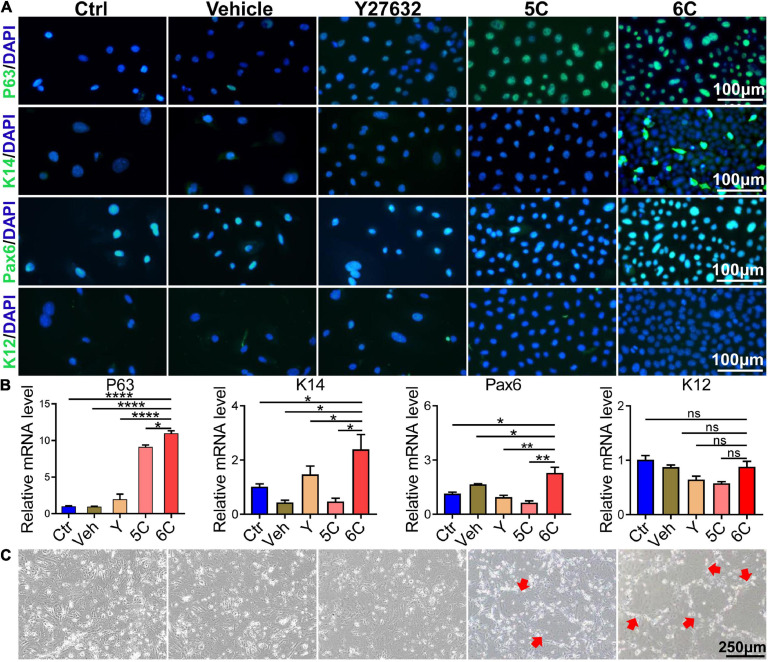
6C can effectively maintain the expression of primary mCEC related genes. **(A)** Immunofluorescence staining of mCEC function markers (green). Nuclei were stained with DAPI (blue). P63 and K14 are markers representing the characteristics of corneal epithelial stem cells. Pax6 is a key transcription factor regulating corneal epithelial cell fate. K12 is a marker of mCEC differentiation. **(B)** qRT-PCR analysis of *P63*, *K14*, *Pax6* and *K12* gene expression (normalized to ctrl). Data expressed as the means ± SEM from three separate experiments (*P < 0.05, **P < 0.01, ****P < 0.0001, ns: no significance). **(C)** 6C can promote the formation of clones of mCEC (red arrow).

Immunofluorescent staining showed that after 6C treatment the P63 and K14 expression levels increased, which is indicative of more abundant proliferating epithelial cell types (Guo et al., 2018; Figure 3A). Such increases were consistent with rises in the Pax6 expression levels, which is another corneal epithelial specific marker (Kitazawa et al., 2019), whereas expression of the differentiation marker, K12, was only marginally expressed in each group. The results of qRT-PCR analysis showed that the increases in the mRNA level of each interrogated gene was consistent with their corresponding protein expression levels (Figure 3B). In addition, we also conducted a 3T3-clone formation assay and found that 6C treatment significantly promoted the formation of cloned cells, which also shows that the cells treated with 6C possess a phenotype more characteristic of proliferating progenitor cells (Figure 3C).

### 6C Treatment Inhibits Spontaneous and FBS-Induced EMT

**FIGURE 4:**
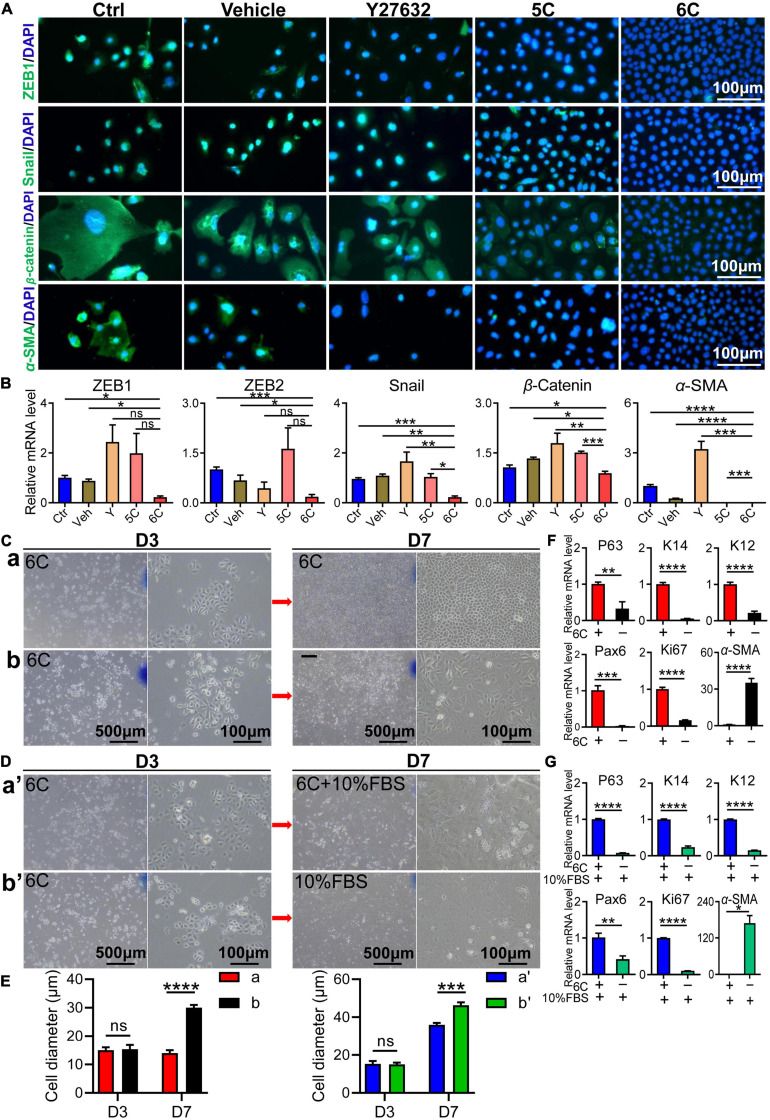
6C can suppress the occurrence of EMT. **(A)** Immunofluorescence staining of EMT-related genes after 1 week of cell culture. ZEB1 and Snail are transcription factors of EMT, β-catenin and α-SMA are markers of EMT (green). Nuclei were stained with DAPI (blue). The expression of EMT-related genes in group 6C was significantly reduced. **(B)** qRT-PCR analysis of *ZEB1/2*, *Snail*, β-*catenin* and α-*SMA* gene expression (normalized to ctrl) after 1 week of cell culture. **(C)** Comparison of cell morphology. **a**, Treat with 6C for 1 week. **b**, Treat with 6C for 3 days, then remove 6C and continue to cultivate for 4 days. **(D)** Comparison of cell morphology. **a’**, It was first treated with 6C for 3 days, and then cultured with 6C plus 10% FBS for another 4 days. **b’**, Treat with 6C for 3 days, then remove 6C and continue to incubate with 10% FBS for 4 days. **(E)** Statistical analysis of cell diameter after C and D treatment. **(F)** After C treatment, qRT-PCR analysis of *P63*, *K14*, *K12*, *Pax6*, *Ki67*, and α-*SMA* gene expression (normalized to 6C). **(G)** After D treatment, qRT-PCR analysis of *P63*, *K14*, *K12*, *Pax6*, *Ki67*, and α-*SMA* gene expression (normalized to 6C plus 10% FBS). Data expressed as the means ± SEM from three separate experiments (*P < 0.05, **P < 0.01, ***P < 0.001, ****P < 0.0001, ns: no significance).

As the cell culture condition cannot mimic the unique microenvironment in the niche supporting stem cell activity *in vivo*, the *in vitro* cell types do not correspond to those proliferating *in vivo* even though there are no definitive biomarkers of stem cells. *In vivo* in this niche there are few cells undergoing an EMT whereas *in vitro* they are easily transformed into mesenchymal cells. Nevertheless, *in vitro* 6C treatment significantly down-regulated this transition because ZEB1 and Snail immunofluorescence were both significantly lower than their levels in the other four groups (Figure 4A). Corresponding measurements of the declines in the mRNA expression levels of *ZEB1*/*2* and *Snail*, which are EMT transcription factors (Tiwari et al., 2017), also show that 6C treatment caused their levels to decline relative to those in the four other treatment groups (Figure 4B). These effects are consistent with the declines in β*-catenin* and α*-SMA* expression levels, which are other characteristic markers of this cell type transition. These results effectively illustrate that 6C treatment significantly inhibited the EMT and thereby plays an important role in maintaining the proliferating phenotype of mCEC.

We determined if 6C treatment suppressed FBS-induced EMT in cultured mCEC. Following exposure to the 6C cocktail for 3 days, the cell confluence had reached about 40% (Figures 4Ca,b). At this time, the cell morphology was regular, and their volume was small (Figure 4E). In one group, 6C treatment was terminated and the culture was continued until four more days had passed (Figure 4Cb). In this group the nuclear and cell volumes enlarged (Figure 4E) whereas in another group in which 6C treatment was continued, they remained very small (Figure 4Ca). The effects were evaluated of serum following 3 days of 6C treatment on cell growth and differentiation (Figures 4Da’,b’). The cell volumes had increased in both the 6C supplemented with 10% FBS and the other group supplemented only with 10% FBS (Figure 4E). However, the degree of differentiation of cells treated with only 10% FBS was pronounced because, the cell boundaries were blurred, and it was difficult to recognize the morphology of individual cells. The qRT-PCR results showed that the 6C-treated group had higher expression levels of progenitor cell markers *P63* and *K14*, differentiation markers *Pax6* and *K12*, as well as higher *Ki67* expression (Figures 4F,G). On the other hand, in the 6C-treated group, α*-SMA* expression was lower, which is an EMT biomarker. Similarly, in the 6C supplemented with 10% FBS group, α*-SMA* expression was lower than that in the 10% FBS treated group. These results substantiate the notion that 6C treatment promoted increases in cell proliferation and inhibited EMT.

### Effects of 6C Treatment on Long-Term Maintenance of mCEC Morphology and Function

**FIGURE 5:**
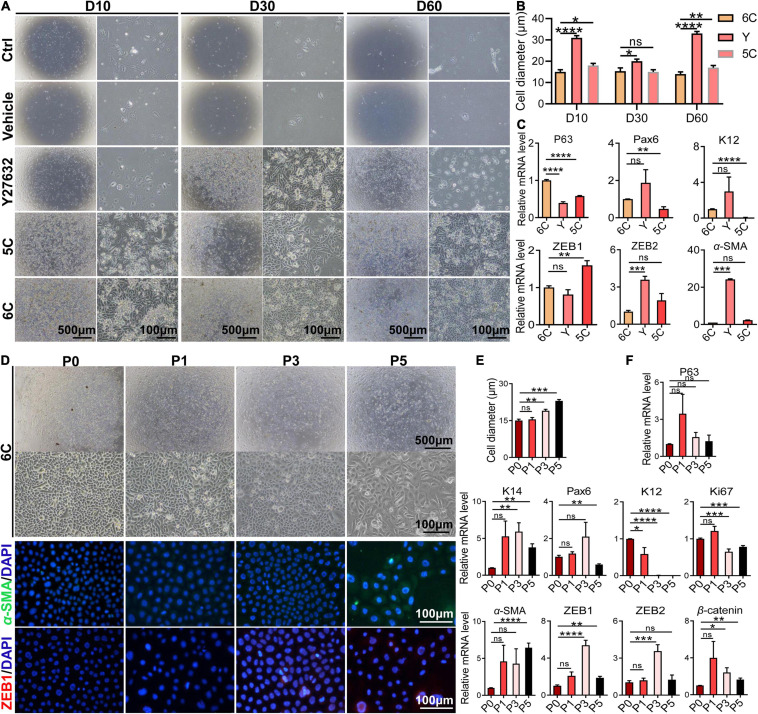
6C can maintain the morphology and function of mCEC in long-term culture and subculture. **(A)** Morphological comparison of mCEC cultured with different compositions in long-term culture. **(B)** Statistical analysis of the diameters of long-term cultured cells. **(C)** qRT-PCR analysis of *P63*, *Pax6*, *K12*, *ZEB1/2*, and α-*SMA* gene expression (normalized to 6C). **(D)** Morphology of mCEC subculture. **(E)** Statistical analysis of the diameters of the cells. **(F)** qRT-PCR analysis of EMT-related genes, mouse corneal epithelial function-related genes and cell proliferation marker *Ki67* (normalized to P0). **(G)** Immunofluorescence staining of EMT-related genes α-SMA (green) and ZEB1 (red). Nuclei were stained with DAPI (blue). Data expressed as the means ± SEM from three separate experiments (*P < 0.05, **P < 0.01, ***P < 0.001, ****P < 0.0001, ns: no significance).

To determine whether 6C protects cells from undergoing declines in proliferative activity and changes in phenotype during an extended period in culture, they were cultured for 60 days. Initially, the microscopic images reveal that the control group and the vehicle group were poorly adherent and weakly proliferative (Figure 5A). After additional time, the cells gradually detached and died. The development of the proliferating status was delayed in the Y-27632-treated group in the first 10 days, but later it was transiently enhanced. By the 60th day, their growth activity had markedly declined and structural integrity became less distinct. The 5C-treated group had the second fastest proliferation rate, which was only exceeded by the 6C-treated group. After culturing the 6C-treated group for 60 days, the interconnections between cells were tight and regular and their apparent cell volumes remained invariant from those at earlier times (Figure 5B). The results of qRT-PCR showed that the expression levels of *P63*, *Pax6*, and *K12* in group 6C were significantly higher than that in the Y-27632 and 5C-treated groups, and the expression of *ZEB1/2* and α*-SMA* in group 6C was significantly lower on the 60th day of culture (Figure 5C). These results substantiate that 6C can effectively maintain mCEC proliferative activity and reduce losses in their integrity resulting from transitioning into a cell type expressing EMT markers.

Next, we further explored the effects of 6C treatment on primary mCEC subcultures. At an initial stage, the mCEC were oval, and as the number of passages increased some cells acquired a long spindle shape (Figure 5D), and their volume increased (Figure 5E). At the mRNA expression level, the expression of the progenitor cell marker *P63* did not change, but the expression of *K14* was up-regulated (Figure 5F). The expression of the transcription factor *Pax6* was down-regulated by the fifth passage. The differentiation marker *K12* was significantly down-regulated. The cell proliferation marker *Ki67* was down-regulated from the third generation. At the same time, as the number of passages increased, the expression levels of EMT-related genes α-*SMA*, *ZEB1/2*, β-*catenin* were up-regulated. By the fifth generation, α-SMA immunofluorescent staining became evident and ZEB1 expression already appeared in the third generation (Figure 5G). The structural integrity and functional activity were only initially preserved of these proliferating cell types following treatment of the mCEC subcultures with 6C. At later times, 6C treatment failed to provide long term protection since the mCEC subcultures appeared to gradually become more similar to other cultures not treated with 6C.

### 6C Treatment Maintains Mouse Corneal Tissue Cultures

**FIGURE 6:**
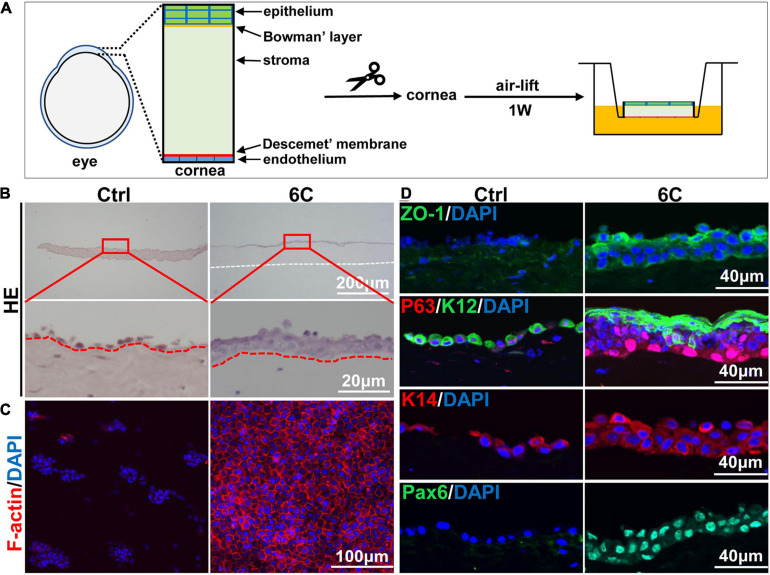
The effect of 6C on mouse cornea *ex vivo* culture. **(A)** Schematic diagram of air-exposed culture of mouse cornea. **(B)** HE staining results of mouse cornea after air exposure culture. The epithelium in the 6C treatment group was relatively intact, while the control group had severe shedding (the white dotted line represents the edge of the corneal stroma and the red dotted line represents the boundary between the epithelial layer and the stromal layer). **(C)** Wholemount immunostaining of F-actin (red). Nuclei were stained with DAPI (blue). **(D)** Immunofluorescence staining of ZO-1 (green), P63 (red), K12 (green), K14 (red) and Pax6 (green). Nuclei were stained with DAPI (blue).

Freshly isolated mouse corneal tissues were air lifted and cultured to simulate the *in vivo* conditions in which the normal cornea maintains itself (Figure 6A). H&E staining results showed that the control groups underwent severe epithelial shedding leaving remaining less than one layer of epithelial cells (Figure 6B). In contrast, the 6C treatment group had developed a relatively complete full thickness stratified epithelium. Furthermore, the distribution of F-actin in the 6C treatment group was regular in these cultures, while the control group had scant levels F-actin expression (Figure 6C). In the 6C treatment group, tight junction protein ZO-1 was expressed in the entire epithelium, but was almost absent in the control group (Figure 6D). In the 6C treatment group, mouse corneal epithelial progenitor cell marker P63 was strongly expressed in the basal and middle part of the epithelium. The mouse corneal epithelial differentiation marker K12 was expressed at the epithelial surface, while the control group had weaker expression of P63 and K12. K14 was used as a limbal progenitor cell marker to further characterize the effect of 6C treatment cocktail on the behavior of corneal epithelial cells in culture (González et al., 2019). Both K14 and the epithelial differentiation regulator Pax6 were almost present throughout the entire epithelium in the 6C treatment group, while only K14 was expressed in the control group. These results show that 6C improves maintenance of the morphology and function of the mouse cornea cultured *ex vivo*.

### 6C Treatment Facilitates the Construction of Tissue Engineered Mouse Corneal Epithelial Sheets

**FIGURE 7:**
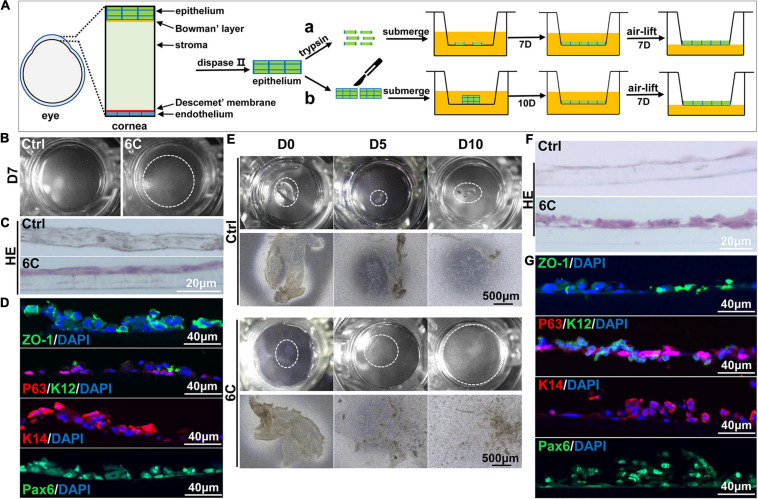
6C helps to construct tissue engineered mouse corneal epithelium. **(A)** Schematic diagram of tissue engineering mouse corneal epithelial sheet culture. **(a)** The isolated mouse corneal epithelium was digested into single cells with trypsin, and then planted into insert. After the cells were confluent, they were cultured in air exposure. **(b)** Divide the isolated corneal epithelium into two and plant it on 12-well cell culture inserts. After the cells are covered with the insert, do air exposure culture. **(B)** When epithelial single cells were cultured to the seventh day, the cells in the 6C treatment group were almost confluent. **(C)** HE staining results of mCEC after air exposure culture. The cells in the 6C treatment group were stratified to two layers. **(D)** Immunofluorescence staining of Z0-1 (green), P63 (red), K12 (green), K14 (red) and Pax6 (green). Nuclei were stained with DAPI (blue). **(E)** Morphological comparison of corneal epithelial sheets cultured with corneal epithelium. In the 6C treatment group, during the culture of the epithelial sheet, the epithelial cells crawled out from the edge, and the surface cells gradually fell off. By the tenth day of culture, the cells were almost confluent, but the epithelium of the control group hardly grew. **(F)** HE staining results of corneal epithelium after air exposure. **(G)** Immunofluorescence staining of Z0-1 (green), P63 (red), K12 (green), K14 (red) and Pax6 (green). Nuclei were stained with DAPI (blue).

As 6C treatment improved the growth and decreased terminal differentiation of corneal explants, we determined if these two effects alter the outcome of tissue engineered mouse corneal epithelial sheets generated for corneal reconstruction surgery. Trypsin was used to detach the mouse corneal epithelium, which was then layered on a Transwell^®^ membrane (Figure 7A). One week later, the cells had nearly completely covered the membranes in the 6C treatment group. However, the cells were poorly adherent in the control groups not treated with the 6C cocktail (Figure 7B). Then both the control and the 6C-treated groups were air-lifted for 1 week to characterize the mCEC differentiation process. The results of H&E staining showed that the cells in the 6C treatment group were stratified to about two layers (Figure 7C). ZO-1 was nearly expressed throughout an entire constructed mouse corneal epithelial sheet (Figure 7D). P63 was expressed in the basal layer of a cell sheet. K14 and Pax6 were expressed in almost all layers. K12 was expressed in the surface layer. The effects of 6C treatment indicate that it is also beneficial to construct tissue-engineered mouse corneal epithelial sheets with a small amount of mouse corneal epithelium (Figures 7E–G). The epithelial sheets constructed by the former method have tighter connections between cells because of the higher expression levels of ZO-1. The expression levels of other genes are almost the same between the two methods.

### Effect of 6C Treatment on Mouse Corneal Epithelial Wound Healing Duration

**FIGURE 8:**
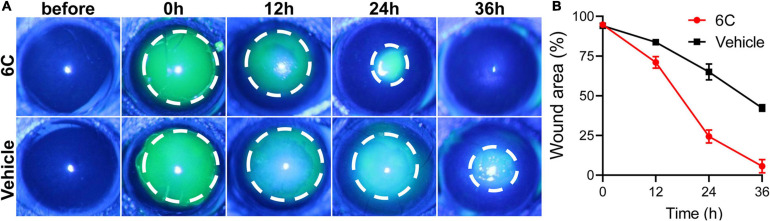
The effect of 6C on wound healing of corneal epithelium in mice. **(A)** Fluorescein sodium nodded and photographed with a slit lamp. 6C can significantly promote wound healing. **(B)** Statistical analysis of wound healing.

Treatment with eye drops containing the 6C cocktail significantly shortened the wound healing time in the corneal epithelium scraping model (Figure 8A). Twenty-four hours after epithelial scraping, the remaining wounded area of the corneal epithelium in the 6C treatment group was 25% of that in the control group (Figure 8B). By the 36th hour, the wound in the 6C treatment group had healed completely, while the corneal epithelium in the solvent group still had a large area defect of 48%. The improved healing of corneal epithelial wounds in the 6C is likely attributable to larger increases in cell proliferation and cell migration in mice (Kao, 2020).

## Discussion

Corneal epithelial renewal and maintenance of function is supported by preservation of different niches whose microenvironment is needed to support different cell phenotypes (Schermer et al., 1986; Ang et al., 2004). This continuous renewal process maintains a functional epithelial layer that supports normal vision through acting as a barrier against pathogenic infiltration. Specifically, the niche in which the slow cycling stem cells reside in the limbus is different from those that promote their stepwise transition to proliferating progenitor cells and differentiating suprabasal cells. The coordination and control of the program that directs this renewal process is very complex and is known to involve a host of different cytokines controlling different steps in this renewal process as well as other factors involved in controlling the expression of these unique microenvironments (Schofield, 1978; Keung et al., 2010).

The insight gained from studies delineating the complexity of this regenerative has improved the outcome of corneal epithelial reconstruction surgery. Such progress stems from larger yields of proliferating corneal epithelial cells needed for generating cell sheets used to resurface the corneal epithelium. The current study is instructive because it identifies a novel procedure for enhancing in a corneal epithelial cell culture the yields of proliferating corneal epithelial cells. This was achieved by treating them with cocktails of different cell signaling modulators which extend their proliferative lifetimes through altering signaling events inducing terminal differentiation and cell death (Xiang et al., 2019). Specifically, the cocktails contained six common inhibitors which reduced the constraints of cell expansion by perpetuating their proliferative status as progenitor surface adherent cells and presumably blocking signals that trigger them instead to undergo declines in cell vitality and undergo terminal differentiation as the cell density increases on the inserts.

It is difficult to maintain for extended periods growth and differentiation of corneal epithelial cells *in vitro.* One of the constraints stems from the loss of the regulatory effect of the *in vivo* microenvironment signals. They are essential to preserve the proliferative status of progenitor cells and prevent the cells from entering into a differentiating phase resulting in EMT and termination of cell expansion (Thiery and Sleeman, 2006; Tiwari et al., 2017). EMT is induced by increases in the expression of a group of transcription factors including members of the Snail, ZEB families and key markers such as β-catenin, α-SMA (Kato et al., 2007; Aomatsu et al., 2011; Lamouille et al., 2014; Puisieux et al., 2014; Tiwari et al., 2017). During this process, losses occur in cell-to-cell coupling in polarized epithelial cells as they undergo transformation into very stable mesenchymal-like cells (Shibata et al., 2019).

Interestingly, our novel 6C cocktail inhibited the expression of spontaneous EMT-related genes and curtailed both this transition and FBS-induced stimulation of this process. Moreover, 6C treatment rendered changes in the cells morphology resulting in their appearance becoming more regular due to increases in the expression levels of the cytoskeletal proteins and tight junctional proteins at the cell membrane surface. These changes resulted in the formation of a continuous belt encircling the cells (Ban et al., 2003), resulting from improved tight junction structural integrity (Anderson, 1977; Sugrue and Zieske, 1997; Ko et al., 2009). Such suppression of EMT was described in other cell types *in vitro*, as follows. Forskolin’s modes of action stems from: reducing EMT possibly due to its activation of cAMP linked signaling pathways, which are known to promote rat corneal epithelium secretion of mucin-like glycoprotein (Nakamura et al., 1998). It is suggested that this response thereby protects cells from external stimulation by cytokines and lubricates their cell surface (Devine and McKenzie, 1992; Cone, 2009; Baudouin et al., 2019). SB431542 is a selective and potent inhibitor of the TGF-β/Activin/NODAL pathway that promotes the differentiation of human induced pluripotent stem cells into corneal epithelial-like cells by restoring the level of endogenous BMP signaling (Kamarudin et al., 2018). DAPT prevents EMT in cultured limbal epithelial stem cells and corneal endothelial cells by inhibiting the Notch signaling pathway (Li et al., 2013; Tsai et al., 2014). IWP-2 is one of the Wnt/β-catenin signaling inhibitors, which can affect the differentiation of human limbal epithelial progenitors (Lee et al., 2017). LDN- 193189 inhibits the BMP signaling pathway, mediating the differentiation of pluripotent stem cells into retinal pigment epithelial cells (Choudhary et al., 2017). Y-27632 is a Rho/Rock signaling pathway inhibitor that promotes corneal limbal cell epithelial proliferation and wound healing (Chen et al., 2008; Yin and Yu, 2008; Miyashita et al., 2013; Sun et al., 2015). It is conceivable that these agents have overlapping inhibitory modes of action since omission of one of them in the previously described 5C cocktail, namely Y-27632, markedly reduced the inhibitory efficacy of the novel 6C cocktail on EMT and generation of proliferating epithelial cells. The 6C cocktail effectiveness in inhibiting EMT was presumably greater than that of the 5C cocktail through larger upregulation of characteristic progenitor genes (P63 and K14) and Pax6. Therefore, the 6C cocktail under serum-free and trophoblast-free conditions is an effective short term preserver of the mouse corneal epithelium linage (Ouyang et al., 2014).

Even though the long-term cultivation of bovine corneal endothelial cells *in vitro* increases their cell surface area and cell size (D’Hondt et al., 2009), in our case 6C culture treatment was needed to extend the short-term proliferative potential of mCEC and the maintenance of tissue-specific cell phenotype. In addition, we used 6C culture conditions to subculture mCEC. As the number of culture passages increased, the progenitor P63 and K14 cell markers continued to be expressed. Their perpetuation has an important regulatory role in maintaining the self-renewal of corneal epithelial cells *in vivo* and can expedite repair of damaged epithelium. This outcome is supported by the fact that their uniform morphology was better maintained than in a basic serum-free medium. Nevertheless, the expression of EMT-related genes rose as the number of passages increased.

It was reported that a permanent rabbit corneal epithelial cell line was established by long-term continuous passage culture on a 3T3 feeder layer (Castromunozledo, 1994). Kawakita also established the mouse corneal epithelial cell line TKE2 using the extended culture method (Kawakita et al., 2008). There are many reports showing that karyotype changes occur with this methodology used to establish an immortalized cell line during culture (Kahn et al., 1993; Skopinski et al., 1998; Jozwiak et al., 2001). Although the 6C culture conditions can maintain the phenotype of mCEC in short-term subculture, their phenotypes may also be unstable due to karyotype changes after multiple passages.

As it was possible to culture mCEC with 6C supplementation, we explored the effect of 6C on native corneal tissue stability *ex vivo*. Furthermore, single cells or small-area epithelial sheets were used to construct functional tissue-engineered corneal epithelial sheets. There are many reports about the *in vitro* culture of human and rabbit limbal tissues (Qi et al., 2008; Miyashita et al., 2013; Suárez-Barrio et al., 2019), but it is still very difficult to culture the mouse corneal epithelium. Human corneal epithelial cells normally express K12 when cultured *in vitro*, but K12 expression is barely detectable in mouse corneal epithelial cells. Even though K12 is rarely expressed in submerged cultured cells, it continues to be expressed in air-lifted corneal tissues and engineered epithelial sheets. This difference indicates that the 6C culture system maintains the progenitor potential of epithelial cells and subsequent air lifting of the cultures effectively simulates the differentiation conducive environment *in vivo* and accordingly promotes cell differentiation. These findings are likely to be insightful in identifying other novel procedures for controlling the balance between mouse corneal epithelial proliferating progenitor and differentiating cells. In addition, *in vivo* experiments have shown that 6C can promote wound healing by stimulating the rapid proliferation of epithelial cells, which may be achieved by maintaining the limbal proliferating stem and progenitor cell phenotype (Li et al., 2007).

Limbal stem cell deficiency can lead to the development of various ocular surface dysfunctions and even blindness. To treat this disease, limbal stem cell transplantation is an effective method to treat ocular surface diseases and reconstruct corneal epithelial structure (Jackson et al., 2020). Our 6C culture system provides novel insight on the identity of targets whose modulation provides both a feasible approach for the *in vitro* expansion of limbal cells and even limbal tissues. Its implementation may ultimately improve viable corneal epithelial cell availability for corneal epithelial reconstruction surgery transplantation in a clinical setting.

## Data Availability Statement

The original contributions presented in the study are included in the article/supplementary material, further inquiries can be directed to the corresponding authors.

## Ethics Statement

The animal study was reviewed and approved by the Experimental Animal Ethics Committee of Xiamen University.

## Author Contributions

CL and YX conceived and designed the experiments. XA performed the research, and collected and analyzed the data. GW, MJ, XZ, SG, JC, and ZL analyzed the data. CL, XA, and PR wrote the manuscript. All authors read and approved the final manuscript.

## Conflict of Interest

The authors declare that the research was conducted in the absence of any commercial or financial relationships that could be construed as a potential conflict of interest.
